# Early life environmental exposures have a minor impact on the gut ecosystem following a natural birth

**DOI:** 10.1080/19490976.2021.1875797

**Published:** 2021-02-02

**Authors:** Nishat Tasnim, Candice Quin, Sandeep Gill, Chuanbin Dai, Miranda Hart, Deanna L. Gibson

**Affiliations:** a Department of Biology, Okanagan Campus, Canada; b Department of Medicine, Faculty of Medicine, University of British Columbia, Kelowna, Canada

**Keywords:** Environment, soil, gut microbiome, immunity

## Abstract

A growing body of evidence suggests that the environment is an important source of colonizing bacteria for the gastrointestinal tract of C-section delivered infants, who undergo multiple birth-related interventions; however, the extent to which environmental microbes impact vaginally delivered infants remains unclear. Here we investigated the impact of rural and urban environmental exposures on microbial establishment and immunity in vaginally delivered mice. We simulated rural and urban home environments by adding soil types to cages from breeding to weaning. Our aims were to determine the impact of rural and urban soil exposures on the gut microbiome in young mice and to understand whether these changes persisted into adulthood. Host immune cytokines and microbial short-chain fatty acids were quantified to understand the impact on immunity. We found that early-life soil exposure had a minor effect on the richness of the neonatal gut microbiota contributing 5% and 9% variation in the bacterial community structure between mice during early-life and adulthood, respectively. Exposure to urban soil increased Clostridiaceae and propionic acid which persisted into adulthood. While soil exposure had a limited effect on the gut taxa, systemic cytokine and chemokine profiles were altered in adulthood. The findings presented here show that unlike in C-section deliveries previously reported, environmental exposures following a natural birth have a limited impact on the gut microbial taxa but potentially play an important role in immune-mediated disease susceptibility later in life.

## Introduction

The early-life microbiome plays a critical role in health outcomes such as metabolism, intestinal homeostasis and physiology, immune maturation and tolerance.^1^ Disruptions in the early microbiome may result in an imbalance in host-microbe equilibrium, termed dysbiosis, which has been associated with health consequences later in life.^2–4^ Although dysbiosis has several potential sources, mode of delivery is commonly cited. During labor and delivery, neonates are exposed to dense bacterial communities. Vaginally delivered infants are largely colonized by maternal vaginal bacterial communities whereas Cesarean sectioned infants acquire skin-like microbes,^5^ found in the operating room.^6^ The corresponding difference in pioneering bacteria caused by C-section delivery has been associated with increased risk for conditions such as inflammatory bowel disease, leukemia, food allergies, obesity and type 1 diabetes. Therefore, the microbial presence in the operating room environment is thought to be an influential factor contributing to dysbiosis in infants delivered by C-section. While the birth environment may contribute to dysbiosis, one criticism against these findings is the other interventions around the time of birth are a major confounding factor. Mothers who deliver by C-section undergo multiple birth-related interventions including antibiotic prophylaxis. While opportunistic pathogens associated with the hospital environment are common in babies delivered by C-section, these microorganisms were also isolated from vaginally delivered infants whose mothers underwent antibiotic prophylaxis or were not breastfed.^7^ It is therefore difficult to disentangle the impact of the hospital environment from other hospital interventions.

Combellick *et al*. addressed this issue by examining fecal microbiota differences in vaginally delivered infants born in the hospital compared to those born at home, in the absence of all interventions.^8^ They found that the microbiota in exclusively breastfed infants differed between the two cohorts, demonstrating a role for the environment in vaginally delivered infants. Despite this important finding, relatively little has been done to characterize environmental microbes and early-life environment remains an overlooked aspect of microbial establishment in vaginally delivered babies.

There are two prominent environmental settings which may differentially impact early-microbial establishment in vaginally delivered infants, including rural and urban living areas. It has been hypothesized that living in urbanized areas could lead to increased risk of allergy, while living in rural areas during childhood may protect against health conditions.^9^ Yet, the scarcity of data on neonatal microbiome establishment in rural versus urban early-life environments is an issue which currently remains unexplored.

In this study, we address the role of the physical environment in establishing gut microbiota in very early life. Our primary objective was to determine the impact of rural, urban or no soil exposures on the gut microbiome in young (3 weeks old) vaginally delivered C57Bl/6 mice. Our secondary objective was to understand whether these changes persisted into adulthood. Concomitantly, we sought to understand the physiological impact of the early gut microbiome on host health. We hypothesized that in the absence of birth-related interventions common to C-section deliveries, early-life environmental exposures would impact the microbiome-immune axis in vaginally delivered mice.

## Materials and methods

### Soil sites and soil sample collection

**Figure 1.**
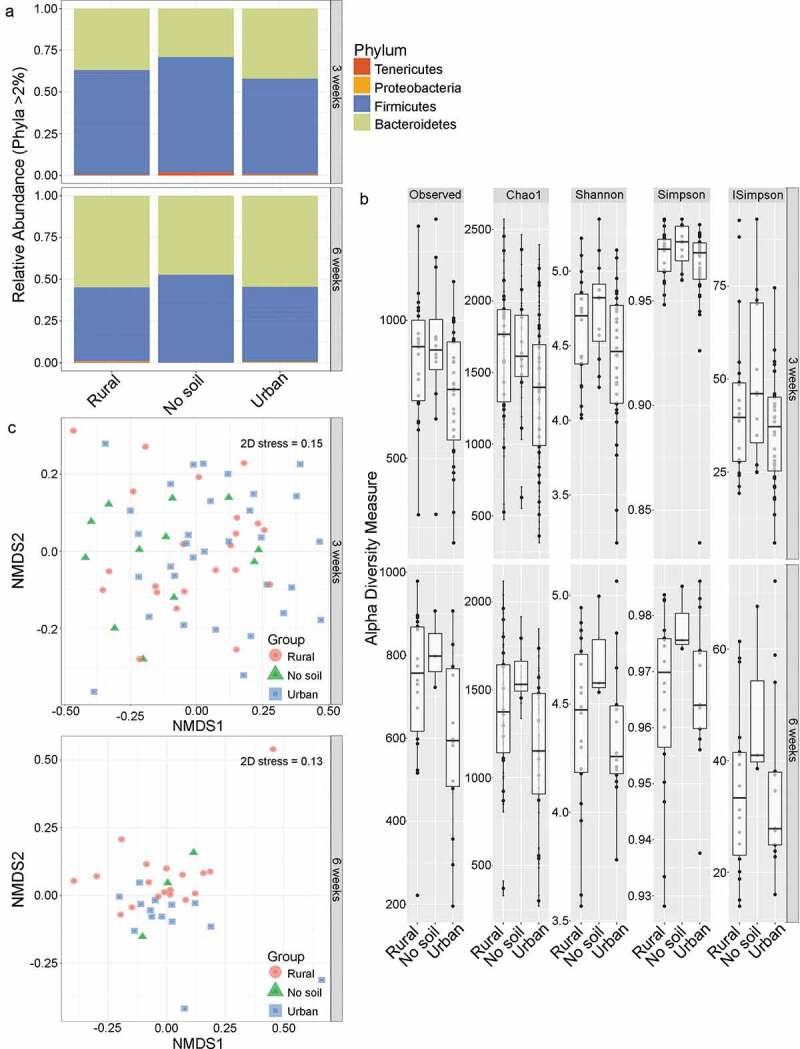
Environmental exposures and gut microbial diversity. A) Relative abundance of gut bacterial phyla of rural soil, urban soil and no soil exposed mice. Each bar represents all the mice exposed to rural soil (n = 23), urban soil (n = 34) and no soil (n = 14) environments at early life (3 weeks) and then aged to 6 weeks under specific pathogen-free conditions. B) Comparison of gut microbiota diversity of early rural soil, urban soil and no soil exposed neonatal and adult mice. Top panel: alpha diversity of neonatal 3 weeks old mice exposed to rural soil (n = 23), urban soil (n = 34) and no soil environments (n = 14). Bottom panel: alpha diversity of 6 weeks old mice kept under regular laboratory conditions after 3 weeks of early exposure to rural soil (n = 20), urban soil (n = 16), and no soil environments (n = 3). Data in box plots represent 25th and 75th percentiles, the lines within the boxes represent the median and the upper and lower whiskers extend from the hinge to the largest and smallest value respectively no further than 1.5*IQR, where IQR is the inter-quartile range, or distance between the first and third quartiles. In each panel, the number of mice is given by circular symbols, which sometimes superimpose on each other. C) The gut bacterial community composition is dissimilar between rural soil, urban soil and no soil exposed groups. Rural soil, urban soil and no soil microbiotas cluster separately, based on two-dimensional non-metric multidimensional scaling (2D-NMDS) of Bray-Curtis dissimilarity distance metric in 3 weeks old neonatal mice (2D stress value = 0.15) and 6 weeks old adult mice (2D stress value = 0.13). Plots show significant clustering of gut microbiota based on early environmental exposure to rural soil, urban soil and no soil environments at 3 weeks of age (Adonis R^2^ = 5.68%, *p* = .041) and 6 weeks of age (Adonis R^2^ = 9.88%, *P* = .061). Ellipses displaying 95% confidence are drawn around exposure groups

Rural soil was collected from a plateau meadow on top of a hill by Chute lake in British Columbia, Canada (GPS coordinates: 49.698859, −119.533133) during the summer season (collection date 24 July 2013). The area where the site is situated is classified under the bio geoclimatic zone: Interior Douglas Fir, dry warm (IDF dw) according to the E-flora BC interactive map based on the provincial Bio geoclimatic Ecosystem Classification (BEC) database (See appendix A Figure 1 for images of soil sites). The majority of the vegetation at the field site consists of Orange hawkweed (*Pilosella aurentiaca*), Hairy vetch (*Vicia villosa*), Oxeye daisy (Leucanthemum* vulgare*), Wild strawberry (*Fragaria virginiana*) Timothy (*Phleum pretense*) and Alsike clover (*Trifolium hybridum*).Urban soil was collected from the parking lot of an Allen and Wright restaurant chain (A&W Restaurants, Inc.) in Kelowna, BC (GPS coordinates: 49.5254, −119.2836) during the summer season (collection date 25 July 2013).

### Animal Care and Experimental Setup

All experimental protocols were approved by the University of British Columbia Animal Care Committee (ACC) at the Center for Disease Modeling (CDM) in Vancouver, Canada. All animal experiments were performed in accordance with the Canadian Council on the Use of Laboratory Animals and ACC guidelines (certificate number A13-0247). C57Bl/6 mice were housed in specific pathogen-free conditions at the CDM and maintained in a restricted access, temperature controlled space (22 ± 2°C) with a 12-hour light cycle. Breeding trios were randomly distributed into three groups: rural soil, urban soil and no soil, and 500 ml of the respective soil was added to sterile cage bedding materials. The soil environment was maintained from breeding throughout weaning, and refreshed during cage changes every 2 weeks. Male and female pups were evenly distributed among treatment groups and were either sacrificed at 3 weeks of age to examine environmental impacts in early-life, or weaned and raised to 6 weeks of age to understand lasting impacts. Weaned mice were co-housed in groups of 3–4 mice per cage and fed a standard pathogen-free diet (Harlan Teklad, cat. #8640) and autoclaved drinking water *ad libitum* (Table S2). This experimental setup was repeated in duplicate and resulted in a total F1 progeny of 43 rural soil pups (20 males and 23 females), 50 urban soil pups (29 males and 21 females) and 17 unexposed pups (10 males and 7 females).

### Tissue Collection

At 3 and 6 weeks of age, blood was collected from the offspring by cardiac puncture during isoflurane anesthesia. Sera was separated from whole blood by allowing the blood to clot undisturbed at room temperature for 15–30 minutes, and then centrifuging at 2,000 x g for 10 minutes at 4°C. Following sacrifice, ileal and colonic tissues were harvested and divided evenly into three segments. The proximal sections were immediately stored in RNAlater (Qiagen; Cat. No. 1018087) to preserve the mRNA prior to homogenization. The distal sections were immersed in 10% neutral buffered formalin (Fisher) and incubated overnight at 4°C. The following day, fixed tissues were washed in phosphate-buffered saline (PBS) solution, and stored in 70% ethanol prior to paraffin embedding and processing at the Child & Family Research Institute (CFRI) histology group, UBC, Vancouver. Lastly, the mid-sections and collected cecal tissues were frozen in liquid nitrogen for high-throughput sequencing and short-chain fatty acid analysis, respectively.

### High-throughput sequencing

DNA was extracted from 5 mm of colonic tissue using QIAamp DNA Stool Mini Kit (QIAGEN, Germany), according to manufacturer’s instructions. Soil DNA was extracted from 125 mg of dry, sieved and homogenized soil following the protocol of the FastDNA-96TM Soil Microbe DNA kit (MP Biomedicals, USA). A “reagent blank” control consisting of all reagents used in the DNA extraction, without the addition of any DNA sample, was used to detect DNA contamination of the analytical reagents, as recommended by previous studies.^10^ After extraction, DNA samples were quantified using Nanodrop^TM^ (Thermo Scientific) and 260/280 readings were recorded to estimate the purity of the DNA. All samples with a 260/280 reading of 1.8 or greater were considered “pure” and amplified in a PCR reaction for the V3-V4 sub-fragment of the 16S rRNA coding region using the 341 f/785 r primer set (5ʹ-ACACTGACGACATGGTTCTACA-3ʹ/5ʹ-TACGGTAGCAGAGACTTGGTCT-3ʹ) . 5ul of each PCR product (~650bp) was run on 1% agarose gel to estimate the band intensity of 16S rRNA gene amplicons. Samples with high intensity bands were then run through a second PCR reaction to attach unique barcodes on the adapter overhang of each sample. Only samples with high intensity bands from the second PCR were sent for Illumina MiSeq sequencing using the 250 paired-end read protocol^11^ at IBEST Genomics Resources Core, University of Idaho. A “no-template” control was used to detect DNA contamination in the amplification reagents.

### Bioinformatics

Quantitative Insights into Microbial Ecology (QIIME)^12^ were used to process raw sequences. Sequences were demultiplexed and quality-filtered using default parameters in QIIME 1.9.1, with an amended quality score cutoff of 19. Operational Taxonomic Units (OTUs) were picked after chimera checking using the recommended open-reference method with 97% identity threshold against Greengenes reference database release 13_8^13^ with USEARCH 6.1^14^ resulting in an average of 14,000 sequences per sample. To correct for uneven library sizes, sequences with counts lower than 943 were filtered out. Taxonomy was assigned by matching the representative sequences clustered to the Greengenes reference file with the corresponding Greengenes id to taxonomy file. The resulting OTU table comprised of 7,333 bacterial taxa from 101 gut samples and 6,866 bacterial taxa from 4 soil samples. This OTU table was used in all downstream statistical analyses.

### Statistical Analyses

All statistical analyses were performed in R version 3.4.0 (R core team 2017) unless otherwise stated. Taxonomic composition was first visualized with stacked bar plots to determine if there were differences in the relative abundance of bacterial phyla between rural, urban and no soil experimental groups at 3 and 6 weeks of age. Only bacterial taxa that were 2% abundant or more across all samples were included.

To compare gut microbiota diversity within 3 and 6 weeks old experimental mice, OTU richness and evenness – alpha diversity – were estimated using Observed, Chao1, Shannon, Simpson and Inverse Simpson diversity indices. Statistical comparisons of alpha diversity indices were performed with a Kruskal–Wallis test and *post hoc* analyses were done with Dunn’s pairwise test using R package PMCMR.^15^ Homogeneity of variance across groups was also computed for each diversity measure with Levene’s test.^16^ Box-and-whisker-plots of each alpha diversity measure were created in R with packages phyloseq^17^ version 1.20.0 and ggplot2^18^ version 2.2.1. Community composition or beta-diversity patterns were analyzed using the Bray–Curtis dissimilarity distance metric. The Bray–Curtis dissimilarity between samples was visualized using two-dimensional non-metric multidimensional scaling (2D-NMDS) analysis to depict community structure patterns in two dimensions. The 2D stress, a measure of how well the NMDS procedure preserves the original rank orders of the OTUs, was calculated to generate plots with low stress. All NMDS plots were generated to have 2D stress <0.2, indicating a good 2D configuration. Bray–Curtis ordination and NMDS plotting were performed with phyloseq and ggplot2 R packages, respectively. To test the hypothesis that cage environments structured the distribution of bacterial communities, permutational multivariate analysis of variance (PERMANOVA) was used with 999 permutations (*P* = .05), computed using Vegan version 2.4–4 *adonis* function.^19^ Additionally, multivariate homogeneity of group dispersion analyses was carried out using vegan v 2.4–4 *betadisper* function to assess the homogeneity of bacterial communities within a group of samples, which could influence PERMANOVA results.^19^

Biomarker identification was performed using linear discriminant analysis effect size analyses (LEfSe)^20^ to determine if any bacterial taxa (OTUs, genera, classes or phyla) were significantly more abundant between rural soil, urban soil or no soil groups at 3 and 6 weeks, using default settings on the website (https://huttenhower.sph.harvard.edu/galaxy/). The alpha value for Kruskal–Wallis and Wilcoxon rank-sum test was set to 0.05, the logarithmic Linear Discriminant Analysis (LDA) score threshold was 2.0, and per sample normalization of sum values was applied (LEfSe default parameters). Bacterial biomarkers identified were microbial taxa that differed in abundance between early environmental exposure groups, as identified by a Wilcoxon rank-sum test. The effect size of each biomarker was then estimated by determining an LDA score. Finally, BugBase^21^ was used to determine if neonatal environmental exposures were associated with any high-level predictive phenotypes. The proportion of aerobic, anaerobic, facultative anaerobic, Gram-positive, Gram-negative, biofilm forming and mobile element containing bacteria were predicted based on the OTU table picked at 97% identity threshold. Statistical analyses of the BugBase predictions were done using a pairwise Mann-Whitney-Wilcoxon test with false discovery rate (FDR) corrections.

### Immunofluorescence

Paraffin-embedded tissue sections were deparaffinized and antigen retrieval was performed on rehydrated tissues using 1 mg/mL trypsin (Sigma-Aldrich, Inc., Darmstadt, Germany) followed by 5% BSA (Sigma-Aldrich) for blocking. Tissues were then incubated with either: rabbit polyclonal antibody raised against myeloperoxidase (MPO) (Thermo Scientific, Waltham, MA) to examine neutrophils (1:400 dilution in 5% BSA); rat monoclonal antibody raised against F4/80 (Cedarlane, Burlington, Ontario) to examine macrophages (1:500 dilution in 5% BSA); rabbit polyclonal IgG antibody-1 raised against CD8 alpha (Abcam, Cambridge, MA). Primary antibody incubation was followed by secondary antibody incubation (1:1000 dilution) with either goat anti-rabbit IgG Alexa Fluor-conjugated 594-red antibody (Invitrogen, Carlsbad, CA) or goat anti-rat IgG HiLyteFluor 488-labeled antibody (Ana Spec Inc., Fremont, CA) or goat anti-rabbit IgG 488-conjugated antibody (Rockland, Limerick, PA). Tissue sections were mounted using fluoroshield with DAPI nuclei stain (Sigma-Aldrich) and viewed on Olympus B51X microscope with Texas Red, FITC and DAPI filters (Olympus Scientific Solutions Americas Corp., Waltham, MA).

Immunofluorescence (IF) stained cells in each colon tissue cross-section were enumerated using manual *in situ* enumeration as previously described.^22^ IF cells in the lamina propria of the colon tissue cross-sections were visualization at 200X magnification by overlaying images captured under fluorescence at an excitation/emission of 350/470 nm for DAPI, 495/519 nm for HiLyteFluor 488 antibody and 590/617 nm for AlexaFlour 594 antibody using MetaMorph Advanced 7.7.8.0 software. For statistical analyses, averages of total cell counts per section of a sample of 5–13 mice from each exposure group were compared using the Kruskal–Wallis rank-sum test using R base stats package and *P* value of less than 0.05 was considered significant.

### Short-chain fatty acid analysis

Short chain fatty acids (SCFA; acetic, propionic and butyric acid) were analyzed in cecal samples using direct-injection gas chromatography (GC) as previously.^23^ The percent mass of SCFA in total cecal sample (% weight SCFA) was calculated as the mg of SCFA per mg of cecal tissue x 100. For statistical comparisons of neonatal and adult mouse %weight SCFA data, two-sample Wilcoxon rank-sum test was performed using R base stats package and *p*-value of less than 0.05 was considered significant.

### Local and systemic cytokine analysis

Thirty-one cytokine/chemokine biomarkers were analyzed from mouse serum by Eve Technologies Corp, Calgary, AB, Canada using a Discovery Assay® (Mouse Cytokine 31-Plex, CAT# MD31). The multiplex assay was performed using the Bio-Plex™ 200 system (Bio-Rad Laboratories, Inc., Hercules, CA), and a Milliplex mouse cytokine kit (Millipore, St. Charles, MO) according to the manufacturers’ protocols. The 31-Plex consisted of tumor necrosis factor alpha (TNF-*α*), IL-1*α*, IL-1*β*, IL-2, IL-3, IL-4, IL-5, IL-6, IL-7, IL-9, IL-10, IL-12 (p40), IL-12 (p70), IL-13, IL-15, IL-17A, interferon-*γ* (IFN-*γ*), macrophage inflammatory protein 1 alpha (MIP-1*α*), MIP-1*β*, macrophage inflammatory protein 2 (MIP-2), regulated on activation, normal T expressed and secreted (RANTES), monocyte chemotactic protein 1 (MCP-1), eotaxin, IFN-gamma-inducible protein 10 (IP-10), lipopolysaccharide-induced CXC chemokine (LIX), vascular endothelial growth factor (VEGF), monokine induced by gamma interferon (MIG), leukemia inhibitory factor (LIF), granulocyte-colony stimulating factor (G-CSF), macrophage colony-stimulating factor (M-CSF), and granulocyte-macrophage colony-stimulating factor (GM-CSF). The sensitivities of these assays ranged from 0.1 to 15.7 pg/mL, and results were expressed as pg/mL of serum. The upper detection limit for each analyte was determined using a standard curve, and the lower detectable concentration was calculated using MILLIPLEX® Analyst 5.1. For IL-13, the upper detection limit was 40,000 pg/mL, and for all other analytes it was 10,000 pg/mL. The minimum detectable concentration varied for each analyte and ranged from 0.5 to 30.6 pg/mL. For statistical comparisons of neonatal and adult mouse secreted serum cytokine concentrations, Wilcoxon rank-sum test was performed using R base stats package and *P* value less than 0.05 was considered significant.

Local colonic cytokines were analyzed by quantitative PCR reactions as described previously.^24^ Briefly, total RNA was purified from 200 mg of homogenized colonic tissue using Qiagen RNEasy kits (Qiagen) according to manufacturer’s instructions. RNA was reverse transcribed with iScript cDNA Synthesis Kit (Bio-Rad) and qPCR was performed using CFX96^TM^ IVD Real-Time PCR Systems equipment (Bio-Rad) and Sso Fast Eva Green Supermix (Bio-Rad). Relative expression of six cytokines (IFN-*γ*, IL-10, TNF-*α*, TGF-*β*, IL-1*β*, and REG-3*γ*) was quantified using CFX Manager software version 1.6.541.1028 (Bio-Rad) where PCR efficiencies for each of the primer sets were incorporated into the final calculations and relative values. All primer sets and efficiencies were previously described and tested.^24^ 18S rRNA was used as a reference gene for gene expression analysis. Relative gene expression was quantified in arbitrary units normalized to 18S rRNA via the ∆∆C_t_ method. For statistical comparison of local cytokine gene expression, the Kruskal–Wallis rank-sum test was performed using R base stats package and *P* value of less than 0.05 was considered significant.

## Results

***Early-life exposure to various environmental exposures does not have an effect on gut microbiome phyla***

Dust is a large component of the indoor exposome, defined as exposures experienced over an individual’s lifespan which affect health. Two-thirds of dust come from soil tracked in from outside, therefore we simulated rural and urban home environments by adding soil either from a rural (forest) or urban (parking lot) soil to the cage bedding materials from the breeding to weaning period. Additional breeding cages that received no soil were used as a proxy for the sterile hospital environment. Our primary objective was to determine the impact of rural, urban or no soil exposures on the gut microbiome in young (3 weeks old) mice. Our secondary objective was to understand whether these changes persisted into adulthood. Concomitantly, we quantified host immune cytokines and microbial short-chain fatty acids (SCFA) to understand the physiological impact of the early gut microbiome on host health and potential underlying mechanisms.

We confirmed that the simulated urban and rural soil environments were distinct (Supplementary Table S1 and Figure S1). The results indicated that the two soil substrates used in the experiment were dissimilar in physical, chemical and microbiological characteristics where the total bacterial richness of rural soil was higher (3,217 OTUs) compared to urban soil (2,818 OTUs), suggesting that the two soils simulated two different soil-enriched neonatal living environments. To understand the effect of the environment on the gut microbiome mice were bred in their particular environment and pups were born and housed in this environment until weaning. Some of these mice were euthanized and tissues examined for gut microbiome at 3 weeks while another cohort of mice were transferred to normal cage environments (no soil) for another 3 weeks to understand if any of the changes see at 3 weeks persisted in the absence of the birth environment into 6-weeks of age. We performed metagenomic sequencing analysis and compared dominant microbial phyla among urban, rural and unexposed experimental groups. The 16S rRNA sequencing data showed that the early environments did not significantly alter the relative abundance of major bacterial phyla between the groups in either the neonatal or adult gut (Figure 1a). The most abundant bacterial phyla dominating the gut microbiota of both neonatal and adult mice were Bacteriodetes and Firmicutes (~97% of total OTUs), irrespective of environmental exposures. In neonatal mice, the most abundant phylum was Firmicutes in all three groups, accounting for 61%, 57% and 69% relative abundance in the rural, urban and no soil cohorts, respectively. In adult mice, the gut microbiota was co-dominated by Bacteriodetes phyla (rural 53%, urban 57% and no soil 44%) and Firmicutes phyla (rural 44%, urban 40%, and no soil 54%). The Tenericutes phylum was specific to neonatal mice and accounted for 1.2–2% relative abundance. In contrast, the gut of adult mice contained Proteobacteria (rural 0.4%, urban 0.9% and no soil 0.1%). Overall, early soil exposure did not significantly alter the phyla composition of the young or adult gut microbiota, suggesting that the early-life external environment has little contribution to the overall taxonomic composition of the infant and mature gut microbiome.

***Early exposure to soil environments transiently altered microbial richness and explained 5–9% of the variation in bacterial community composition***

To assess whether early soil exposures associated with changes in bacterial richness and evenness within gut microbial communities, alpha diversity measures were plotted and compared. We found that alpha diversity estimates were not associated with soil exposure and found no significant impact of environment on Observed, Chao1, Shannon, Simpson and Inverse Simpson diversity indices at 3 or 6 weeks of age (Figure 1b, Table 1). In contrast to alpha diversity, multivariate analyses showed the bacterial community structure, beta diversity, was influenced by the early-life environment and accounted for 5–9% variation among experimental groups at both 3 and 6 weeks age (Figure 1c, Table 2). Results from the PERMANOVA and multivariate dispersion analyses revealed that the microbial community was significantly different among the 3 week old experimental mice despite high variability between individuals (Adonis R^2^ = 5.67%, *P* = .041). These early-life differences in gut bacterial community structures marginally persisted into adulthood (Adonis R^2^ = 9.87%, *P* = .061). Overall, these findings confirm a minor role for environment on gut microbial composition during early life.

**Table 1.** Kruskal–Wallis results of alpha diversity matrices

Metric	Age	Df	Chi-squared	P
Observed	3 weeks	2	6.2506	0.04392*
Chao1	3 weeks	2	5.4014	0.06716
Shannon	3 weeks	2	5.1381	0.07661
Simpson	3 weeks	2	3.402	0.1825
InvSimpson	3 weeks	2	3.402	0.1825
Observed	6 weeks	2	4.7978	0.09082
Chao1	6 weeks	2	4.279	0.1177
Shannon	6 weeks	2	3.1474	0.2073
Simpson	6 weeks	2	2.5776	0.2756
InvSimpson	6 weeks	2	2.5776	0.2756

*indicates statistical significance at *P* < 0.05.

**Table 2.** Permutational multivariate analyses (PERMANOVA) results based on Bray–Curtis dissimilarity distances

*PERMANOVA between the different cage environments*							
**Age (weeks)**	**#perm**	**Df**	**SS**	**MS**	**F.model**	**R**^2^	***P***
3	999	2	0.5150	0.25750	1.8956	0.05676	0.041*
6	999	2	0.2779	0.13895	0.1390	0.09878	0.061
*Multivariate homogeneity of group dispersion analysis between the different cage environments at each age group*							
**Age (weeks)**	**#perm**	**Df**	**SS**	**MS**	**F.model**	***P***	
3	999	2	0.00365	0.0018259	0.2484	0.773	
6	999	2	0.026781	0.0133905	1.3909	0.266	

*indicates statistical significance at *P*< 0.05. Df, degrees of freedom; SS, sum of squares; MS, mean sum squares; F model of permutation; *P*-value based on 999 permutations

***Early-life urban soil exposure significantly increased the differential abundance Clostridiaceae in the infant microbiota which persisted into adulthood***

**Figure 2.**
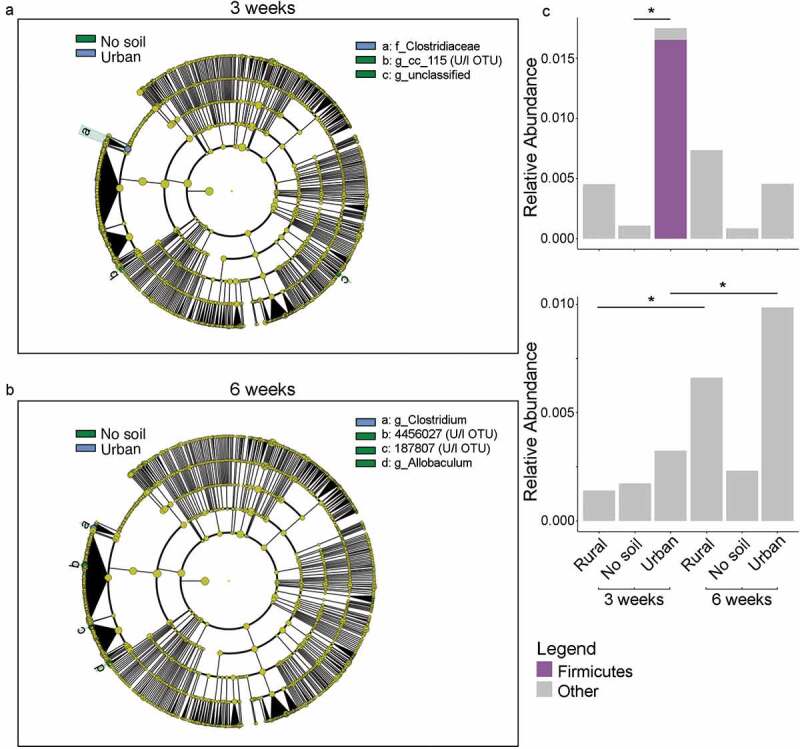
Differentially abundant microbial taxa between mice given early exposure to rural soil, urban soil and no soil. Linear discriminant analyses (LDA) using LEfSe were applied to identify biomarkers at higher taxonomic levels (down to genus level). Samples were compared by A) early exposure at 3 weeks and B) 6 weeks age to identify LEfSe microbial taxa biomarkers. No bacterial biomarkers were identified in 3 weeks old and 6 weeks old mice given early rural soil exposure. Three LEfSe biomarkers were identified at 3 weeks, with one microbial taxa from family (f_) Clostridiaceae that was differentially abundant in mice given early exposure to urban soil (n = 34 [in blue]), and two unidentified microbial taxa, genus (g_) cc_115 and g_unclassified, that were differentially abundant in no soil group (n = 14, [in green]). Four LEfSe biomarkers were identified at 6 weeks, with one microbial taxa from genus (g_) *Clostridium* being differentially abundant in mice given early exposure to urban soil (n = 16), and three biomarkers including genus (g_) *Allobaculum* being enriched in mice raised in a no soil environment (n = 3). BugBase predicted phenotypes of C) aerobic bacteria at 3 weeks (top panel) and biofilm formers at 3 and 6 weeks (bottom panel). Values are displayed as mean OUT contributions. All associations had *P* values of <0.05 after correcting for multiple testing

**Figure 3.**
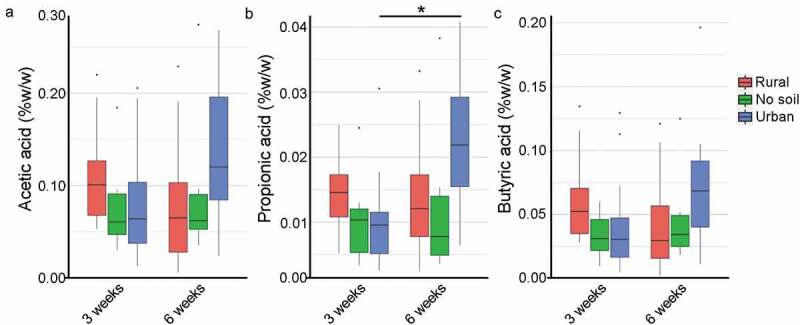
Effect of early soil exposures compared to no soil exposure on relative concentrations of cecal short-chain fatty acids (SCFAs). Cecal SCFA concentrations of acetic acid (a), propionic acid (b) and butyric acid (c) of rural soil, no soil and urban soil exposed mice at 3 weeks and 6 weeks of age (n = 6–12 per age group) are reported as % w/w (cecal tissue) via gas-chromatography (see Materials and Methods). Data in box plots represent 25th and 75th percentiles, the lines within the boxes represent the median and the upper and lower whiskers extend from the hinge to the largest and smallest value respectively no further than 1.5*IQR (where IQR is the inter-quartile range, or distance between the first and third quartiles). In each panel, the number of mice is given by circular symbols, which sometimes superimpose on each other. (Wilcoxon rank-sum test; * *P* = .003)

**Figure 4.**
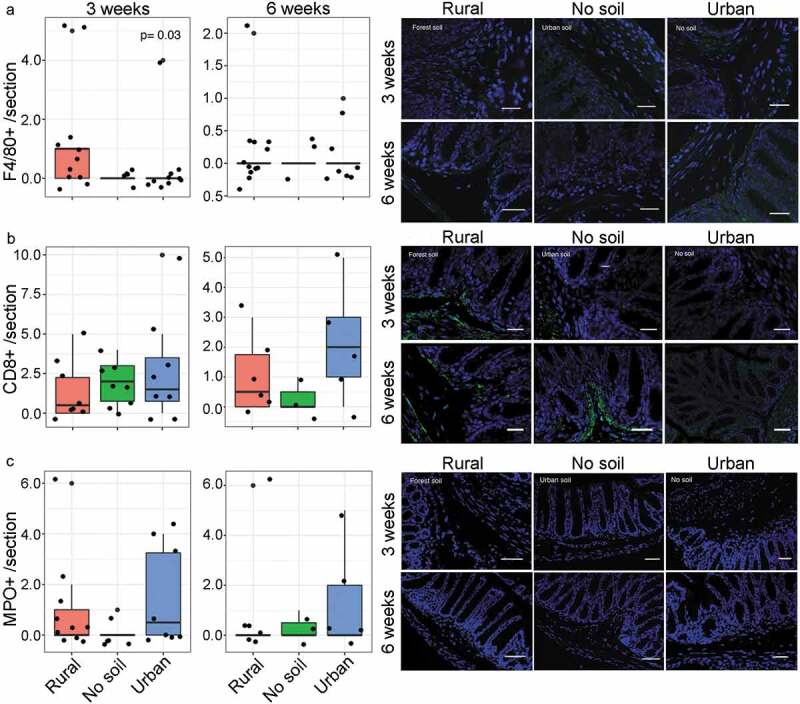
Effect of early rural soil, urban soil and no soil exposure on intestinal immune cell infiltration in neonatal (3 weeks old) and mature mice (6 weeks old).Colon sections were stained for presence of A) submucosal F4/80+ macrophages, B) CD8 + T cells and C) MPO+ neutrophils. In each plot, the number of mice is given by circular symbols (n = 5–13 per group), which sometimes superimpose on each other. Data in box plots represent 25th and 75th percentiles, the lines within the boxes represent the median and the upper and lower whiskers extend from the hinge to the largest and smallest value respectively no further than 1.5*IQR (where IQR is the inter-quartile range, or distance between the first and third quartiles). Early rural soil exposure increased the submucosal infiltration of macrophages in neonatal mice (Kruskal-Wallis, *P* = .03). Representative immunofluorescence images of A) F4/80+ cells using FITC filter, B) CD8+ cells using FITC filter and C) MPO+ cells using Texas Red filter are shown at 200X magnification, with DAPI nuclear counterstain with DAPI filter (scale bar = 14um)

To further investigate differences in community composition among groups, a LEfSe analysis was performed. LEfSe identified three gut microbial taxonomic biomarkers of early environmental exposure in young mice (Figure 2a). The biomarker for early urban soil exposure in young mice was the family Clostridiaceae, and the biomarkers for no soil exposure were unclassified phylotypes. No biomarkers of early rural soil exposure were identified in young mice. In 6 week old adult mice, LEfSe identified four gut microbial taxonomic biomarkers of early environmental exposure (Figure 4b Figure 3). The biomarker for early urban soil exposure in adult mice was the genus *Clostridium*. In contrast, the dirt-free mice were characterized by elevated levels of *Allobaculum* spp. and two unclassified phylotypes. Similar to neonatal mice, no biomarkers of early rural soil exposure were identified in adult mice. Overall, although few bacterial taxonomic biomarkers were associated with early-life exposure to urban soil and no soil cage environments, there was a consistent association with bacterial biomarker family *Clostridiaceae* and early urban soil exposure, indicating that early-life exposure to urban soil may increase the colonization and establishment of gut commensals from family *Clostridiaceae*. We furthered our analysis by assessing whether soil exposures were associated with any predicted high-level phenotypes. The BugBase results revealed that aerobic bacteria belonging to the phylum Firmicutes were increased in the urban soil cohort at 3 weeks of age when compared to the no soil cohort (*P* = .004). However, differences between the urban and no soil cohort did not persist into adulthood and there were no differences between the three experimental groups at 6 weeks of age. There were, however, changes in predicted microbial phenotypes within environmental groups with age. The findings indicated that biofilm forming bacteria increased with age in the rural (*P* = .01) and urban soil (*P* = .05) exposed mice (Figure 2b).

### Early-life urban soil exposure associated with higher production of propionic acid in adulthood

To understand the correlation between microbiota community structure differences and metabolic differences among the exposure groups, we quantified SCFA. SCFA are bacterial metabolites that regulate immune responses and healthy gut physiology, and their production can be altered by changes to the gut microbial community composition.^25^ SCFA analysis revealed no significant differences in levels of acetic acid and butyric acid between 3 weeks and 6 weeks in each exposure group (Figure 3). The production of propionic acid was similar between neonatal and adult mice in rural soil and no soil groups; however, a significant increase in cecal propionic acid production was observed in the urban experimental mice as they aged (*P* = .003). Overall, the SCFA profiles of acetic and butyric acid were relatively unchanged by early environmental exposure in both neonatal and young adult age groups; however, urban soil exposure correlated with an increase in propionic acid in mice at 6 weeks of age, suggesting early urban soil exposure effects intestinal health and immunity as the rodents aged.

### Early rural soil exposure increased colonic macrophage infiltration in young mice

Gut microbiota colonization and maturation play an important role in the development of host immune responses both directly through the establishment of host-microbiota symbiosis and indirectly through the production of bacterial metabolites. Given the alterations in microbial biomarkers, we then examined the effects of soil exposures on intestinal lymphoid cells such as lymphocytes, neutrophils and macrophages/monocytes by quantifying immune cell populations via immunofluorescence (Figure 4). Early rural soil exposure resulted in higher infiltration of F4/80+ macrophages in neonatal mice colonic submucosa compared to urban soil and no soil exposed mice at 3 weeks of age (*P* = .03). This increase in colonic macrophage infiltration did not persist in mature mice as there were no significant differences in macrophage counts between groups at 6 weeks age. Similarly, there were no significant differences in neutrophil infiltration or CD8 + T cell populations in experimental groups at 3 or 6 weeks of age. Colonic cytokine expression also showed no differences in local cytokine responses at either 3 or 6 weeks of age. Taken together, these results indicate that intestinal immunity was not strongly affected by early environmental exposure during infancy; however, there was a notable increase in macrophage infiltration following rural soil exposure suggesting a transient impact on early host immune responses.

***Early exposure to rural soil and urban soil was associated with altered serum cytokine and chemokine concentrations in mature mice***

**Figure 5.**
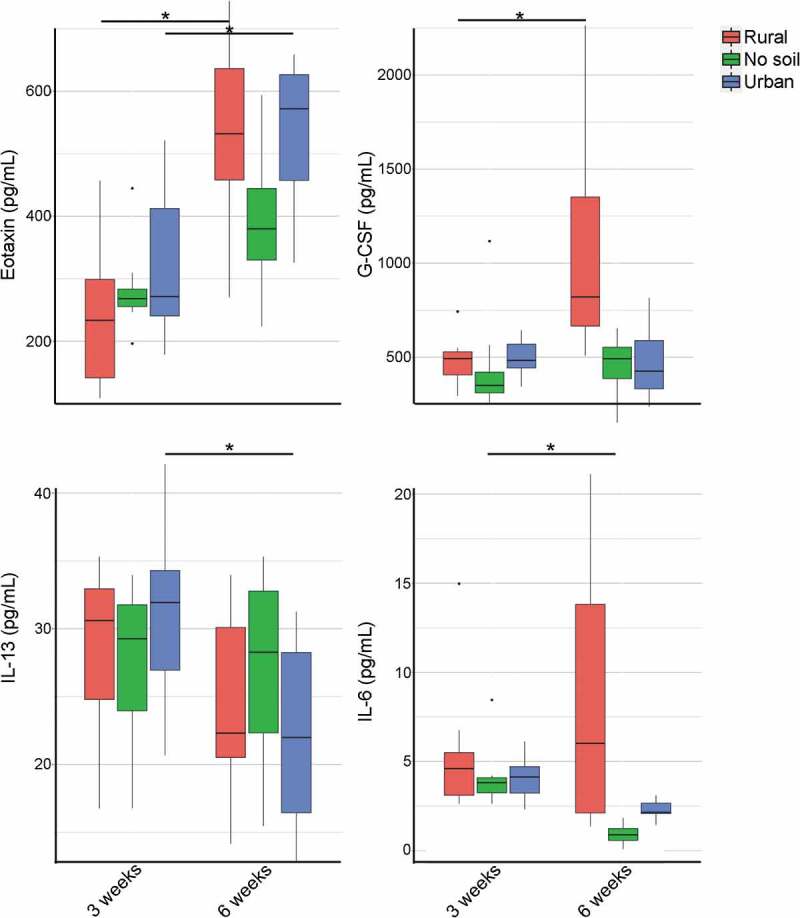
Effect of early environmental exposure to rural soil, urban soil and no soil on serum cytokine and chemokine responses in neonatal and mature mice. Serum cytokine concentrations (pg/mL) were measured using Mouse cytokine/chemokine array 31-Plex (Eve Technologies). The upper detection limit for IL-13 was 40,000 pg/mL, and for all other analytes in the array it was 10,000 pg/mL. Data in box plots represent 25th and 75th percentiles, the lines within the boxes represent the median and the upper and lower whiskers extend from the hinge to the largest and smallest value respectively no further than 1.5*IQR (where IQR is the inter-quartile range, or distance between the first and third quartiles). In each boxplot, the number of mice is given by circular symbols, which sometimes superimpose on each other (n = 3–11 per group). (Wilcoxon rank-sum test; *P < .05)

While gut immunity was mostly unaffected by the early-life environment, to understand if there was any effect on systemic immunity, we quantified serum cytokine responses. Cytokines including IL-1*α*, IL-1*β*, MCP-1, IL-10 and TNF-*α* were similar among experimental groups at 3 and 6 weeks of age, which suggests normal cytokine-driven immunity in all groups of mice. While experimental cohorts did not differ in cytokine profiles, there were select age-associated changes between young and adult mice (Figure 5). Specifically, the serum concentration of the chemokine eotaxin increased between young and adult mice in both rural (*P* = .003) and urban soil experimental groups (*P* = .005). Additionally, the serum concentration of granulocyte-colony stimulating factor (G-CSF) increased with age in the rural soil group (*P* = .015). In contrast, the serum concentration of anti-inflammatory IL-13 was lower in 6 weeks old mice when compared to 3 weeks old mice only in the urban soil group (*P* = .03). Serum concentration of secreted pro-inflammatory cytokine IL-6 also decreased in adult mice compared to neonatal mice in the no soil group (*P* = .01). Besides these four chemokines and cytokines, all other serum cytokine and chemokine concentrations were similar in the different exposure groups at both ages. Taken together, early environmental exposure did not alter systemic cytokine responses in young mice but was associated with some altered systemic cytokine and chemokine profiles as mice aged.

## Discussion

In this study, we investigated the impact of rural and urban environmental exposures on microbial establishment and health outcomes in vaginally delivered C57Bl/6 mice. Since the taxonomic composition of soil bacterial communities vary between biomes,^26^ physical and chemical gradients^27^ and anthropogenic activity,^28^ we first characterized soils collected from rural and urban environments. We found that urban soil had reduced biodiversity, with decreased counts of soil bacteria and smaller hyphal lengths compared with rural soil. This degradation of soil quality with urbanization has been reported previously^29^ and validated using rural-urban soils for creating distinct home milieus.

Despite physical, chemical and microbiological differences in cage environments, changes to the microbiome-immune axis in experimental groups were limited. This is in contrast to the prevailing idea that environment has a major effect on the development of the gut microbiome. Our results found that the gut microbiome was initially dominated by Firmicutes compared to Bacteroidetes in young mice, irrespective of soil group. As mice aged, the phyla Bacteroidetes increased. This shift in microbial composition from Firmicutes dominance in the infant gut microbiota to Firmicutes and Bacteriodetes co-dominance in the mature gut microbiota is well documented^30^ and coincides with what is observed in children under the age of four years. This suggests that soil exposure was not a significant contributor to microbial diversity at the phylum level. Similarly, there were no differences in alpha diversity indices at 3 or 6 weeks of age.

Beta diversity analyses likewise found a limited impact of soil exposure on microbial community composition. Gut communities clustered significantly by experimental group, which accounted for 5–9% of bacterial community variation. By comparison, mode of delivery has been shown to account for up to 21.5% variation.^31^ The bacteria detected were in many cases common vaginal residents, suggesting that the microbiota in experimental groups were derived primarily from maternal exposures during birth.^32^ Nevertheless, distinct LEfSe biomarkers defined different exposures. Notably, mice exposed to urban soil had elevated Clostridiaceae and mice raised in the absence of soil had enriched *Allobaculum* spp. While knowledge of the role of *Allobaculum spp*. is limited and their effect in inflammation remains unclear, Clostridiaceae are often associated with poor health. For instance, an increase in different *Clostridia* class groups has been associated with the development and onset of allergic diseases.^33–35^ The increased abundance of taxa in this family could suggest that urban soil exposure increases the risk for allergy. In support of this notion, we observed increases in systemic eotaxin expression with age in the urban soil cohort, which plays a central role in the pathogenesis of allergic disease.^36,37^ However, comparative eotaxin expression was observed the rural soil cohort which did not have discernable allergy-associated microbial patterns. Additionally, IL-13 expression decreased in the urban soil cohort with age. IL-13 is required for allergic airway responses and promotes isotype switching of B cells to produce IgE. It is therefore unlikely that the urban soil cohort is more at risk for allergy, and the increase in eotaxin expression is likely a natural aging phenomenon.^38^ Furthermore, unlike *C. difficile* which is common in hospital-delivered infants,^39^ the Clostridiaceae that colonized the urban soil cohort were not likely pathogenic, as there were no differences in major cytokine or chemokine expression between experimental groups and there was an increase in propionic acid with age, which is beneficial at appropriate levels. It should be stated, however, that elevated *Clostridia* spp. and propionic acid production has also been observed in patients with autisum spectrum disorders,^40,41^ but no overt behavioral abnormalities were noted in our mice.

The findings from this study suggest that environmental microbes from soil, either from an urban or forested plot, have a minor impact on infant microbial colonization yet impacts basal cytokine production with age. Alterations in the immune aging trajectory may be important in the host’s ability to respond to infectious pathogens or could alter tolerance so that chronic diseases like allergies or autoimmune diseases develop. More research is needed to understand if there are any consequences to any aberrant immune responses due to early-life environment in vaginally birthed babies. Overall, these findings suggest that the influence of environment in a natural birth setting is limited but not negligible. Despite the birth environment eliciting few changes in microbial taxa, the birth environment could impact disease susceptibility later in life including to infectious disease or chronic diseases including allergies and autoimmune disease. Future studies are needed to better understand the physiological impacts of early-life environmental exposures in vaginally delivered infants.

## Supplementary Material

Supplemental Material

## References

[1] Lynch SV, Pedersen O. The Human Intestinal Microbiome in Health and Disease. N Engl J Med. 2016;375:2369–15. doi:10.1056/NEJMra1600266. 27974040

[2] Thavagnanam S, Fleming J, Bromley A, Shields MD, Cardwell CR. A meta-analysis of the association between Caesarean section and childhood asthma. Clin Exp Allergy. 2008;38(4):629–633. doi:10.1111/j.1365-2222.2007.02780.x. 18352976

[3] Marild K, Stephansson O, Montgomery S, Murray JA, Ludvigsson JF. Pregnancy outcome and risk of celiac disease in offspring: a nationwide case-control study. Gastroenterology. 2012;142(1):39–45 e3. doi:10.1053/j.gastro.2011.09.047. 21995948 PMC3244504

[4] Darmasseelane K, Hyde MJ, Santhakumaran S, Gale C, Modi N. Mode of delivery and offspring body mass index, overweight and obesity in adult life: a systematic review and meta-analysis. PLoS One. 2014;9(2):e87896. doi:10.1371/journal.pone.0087896. 24586295 PMC3935836

[5] Dominguez-Bello MG, Costello EK, Contreras M, Magris M, Hidalgo G, Fierer N, etKnight R. Delivery mode shapes the acquisition and structure of the initial microbiota across multiple body habitats in newborns. Proc Nat Acadf Sci USA 2010; 107:11971–11975. 10.1073/pnas.1002601107 PMC2900693 20566857

[6] Shin H, Pei Z, Martinez KA 2nd, Rivera-Vinas JI, Mendez K, Cavallin H,Dominguez-Bello MG. The first microbial environment of infants born by C-section: the operating room microbes. Microbiome. 2015;3(1):59. doi:10.1186/s40168-015-0126-1. 26620712 PMC4665759

[7] Shao Y, Forster SC, Tsaliki E, Vervier K, Strang A, Simpson N,Kumar N, Stares MD, Rodger A, Brocklehurst P, Field N, Lawley TD. Stunted microbiota and opportunistic pathogen colonization in caesarean-section birth. Nature. 2019;574(7776):117–121. doi:10.1038/s41586-019-1560-1. 31534227 PMC6894937

[8] Combellick JL, Shin H, Shin D, Cai Y, Hagan H, Lacher C, Lin DL, McCauley K, Lynch SV, Dominguez-Bello MG. Differences in the fecal microbiota of neonates born at home or in the hospital. Sci Rep. 2018;8(1):15660. doi:10.1038/s41598-018-33995-7. 30353125 PMC6199260

[9] Strachan DP. Family size, infection and atopy: the first decade of the “hygiene hypothesis”. Thorax. 2000;55(Suppl 1):S2–10. doi:10.1136/thorax.55.suppl_1.S2. 10943631 PMC1765943

[10] Sundquist T, Bessetti J Identifying and Preventing DNA Contamination in a DNA Typing-Laboratory. In: Corp. P, ed.: Promega Corp., 2005.

[11] Fadrosh DW, Ma B, Gajer P, Sengamalay N, Ott S, Brotman RM, Ravel J. An improved dual-indexing approach for multiplexed 16S rRNA gene sequencing on the Illumina MiSeq platform. Microbiome. 2014;2:6. doi:10.1186/2049-2618-2-6. 24558975 PMC3940169

[12] Caporaso JG, Kuczynski J, Stombaugh J, Bittinger K, Bushman FD, Costello EK, Fierer N, Gonzalez Peña a, Goodrich JK, Gordon JI, Huttley GA, Kelley ST, Knights D, Koenig JE, Ley RE, Lozupone CA, McDonald D, Muegge BD, Pirrung M, Reeder J, Sevinsky JR, Turnbaugh PJ, Walters WA, Widmann J, Yatsunenko T, Zaneveld J, Knight R. QIIME allows analysis of high-throughput community sequencing data. Nat Methods. 2010;7(5):335–336. doi:10.1038/nmeth.f.303. 20383131 PMC3156573

[13] DeSantis TZ, Hugenholtz P, Larsen N, Rojas M, Brodie EL, Keller K, Huber T, Dalevi D, Hu P, Andersen GL. Greengenes, a chimera-checked 16S rRNA gene database and workbench compatible with ARB. Appl Environ Microbiol. 2006;72(7):5069–5072. doi:10.1128/AEM.03006-05. 16820507 PMC1489311

[14] Edgar RC. Search and clustering orders of magnitude faster than BLAST. Bioinformatics. 2010;26(19):2460–2461. doi:10.1093/bioinformatics/btq461. 20709691

[15] Pohlert T The Pairwise Multiple Comparison of Mean Ranks Package (PMCMR) 2014.

[16] Fox J, Weisberg S. eds. An R companion to applied regression.; 2011.

[17] McMurdie PJ, Holmes S. phyloseq: an R package for reproducible interactive analysis and graphics of microbiome census data. PloS One. 2013;8(4):e61217. doi:10.1371/journal.pone.0061217. 23630581 PMC3632530

[18] Wickham H Ggplot2: elegant Graphics for Data Analysis. In: York S-VN, ed., 2009.

[19] J Bf O, Kindt R, Legendre P, Minchin PR, O’Hara RB, Simpson GL, Solymos P, Stevens MHH, Vegan: WH Community ecology package. R package version 24–34, 2016.

[20] Segata N, Izard J, Waldron L, Gevers D, Miropolsky L, Garrett WS, Huttenhower C. Metagenomic biomarker discovery and explanation. Genome Biol. 2011;12(6):R60. doi:10.1186/gb-2011-12-6-r60. 21702898 PMC3218848

[21] Ward T, Larson J, Meulemanns J, Hillmann B, Lynch J, Sidiropoulos D, Spear JR, Caporaso G, Blekhman R, Knight R, Fink R, Knights D. BugBase predicts organism level microbiome phenotypes. 2017.

[22] Preza GC, Tanner K, Elliott J, Yang OO, Anton PA, Ochoa MT. Antigen-presenting cell candidates for HIV-1 transmission in human distal colonic mucosa defined by CD207 dendritic cells and CD209 macrophages. AIDS Res Hum Retroviruses. 2014;30(3):241–249. doi:10.1089/aid.2013.0145. 24134315 PMC3938918

[23] DeCoffe D, Quin C, Gill SK, Tasnim N, Brown K, Godovannyi A, Dai C, Abulizi N, Chan YK, Ghosh S, Gibson DL. Dietary Lipid Type, Rather Than Total Number of Calories, Alters Outcomes of Enteric Infection in Mice. J Infect Dis. 2016;213(11):1846–1856. doi:10.1093/infdis/jiw084. 27067195

[24] Ghosh S, DeCoffe D, Brown K, Rajendiran E, Estaki M, Dai C, Yip A, Gibson DL. Fish oil attenuates omega-6 polyunsaturated fatty acid-induced dysbiosis and infectious colitis but impairs LPS dephosphorylation activity causing sepsis. PloS One. 2013;8(2):e55468. doi:10.1371/journal.pone.0055468. 23405155 PMC3566198

[25] Morrison DJ, Preston T. Formation of short chain fatty acids by the gut microbiota and their impact on human metabolism. Gut Microbes. 2016;7(3):189–200. doi:10.1080/19490976.2015.1134082. 26963409 PMC4939913

[26] Andriuzzi WS, Wall DH. Responses of belowground communities to large aboveground herbivores: meta-analysis reveals biome-dependent patterns and critical research gaps. Glob Chang Biol. 2017;23(9):3857–3868. doi:10.1111/gcb.13675. 28245090

[27] Fierer N, Leff JW, Adams BJ, Nielsen UN, Bates ST, Lauber CL, Owens S, Gilbert JA, Wall DH, Caporaso GJ. Cross-biome metagenomic analyses of soil microbial communities and their functional attributes. Proceedings of the National Academy of Sciences of the United States of America 2012; 109:21390–21395. 10.1073/pnas.1215210110 PMC3535587 23236140

[28] Lauber CL, Ramirez KS, Aanderud Z, Lennon J, Fierer N. Temporal variability in soil microbial communities across land-use types. Isme J. 2013;7(8):1641–1650. doi:10.1038/ismej.2013.50. 23552625 PMC3721119

[29] Kemper KJ, Lal R. Pay dirt! human health depends on soil health. Complement Ther Med. 2017;32:A1–A2. doi:10.1016/j.ctim.2017.04.005. 28619312

[30] Mariat D, Firmesse O, Levenez F, Guimaraes V, Sokol H, Dore J, Corthier G, Furet JP. The Firmicutes/Bacteroidetes ratio of the human microbiota changes with age. BMC Microbiol. 2009;9(1):123. doi:10.1186/1471-2180-9-123. 19508720 PMC2702274

[31] Reyman M, van Houten MA, van Baarle D, Bosch A, Man WH, Chu M, Arp K, Watson RL, Sanders EAM, Fuentes S, Bogaert D. Impact of delivery mode-associated gut microbiota dynamics on health in the first year of life. Nat Commun. 2019;10(1):4997. doi:10.1038/s41467-019-13014-7. 31676793 PMC6825150

[32] Funkhouser LJ, Bordenstein SR. Mom knows best: the universality of maternal microbial transmission. PLoS Biol. 2013;11(8):e1001631. doi:10.1371/journal.pbio.1001631. 23976878 PMC3747981

[33] Wopereis H, Oozeer R, Knipping K, Belzer C, Knol J. The first thousand days - intestinal microbiology of early life: establishing a symbiosis. Pediatr Allergy Immunol. 2014;25(5):428–438. doi:10.1111/pai.12232. 24899389

[34] Bjorksten B, Sepp E, Julge K, Voor T, Mikelsaar M. Allergy development and the intestinal microflora during the first year of life. J Allergy Clin Immunol. 2001;108(4):516–520. doi:10.1067/mai.2001.118130. 11590374

[35] Marrs T, Flohr C. How do Microbiota Influence the Development and Natural History of Eczema and Food Allergy? Pediatr Infect Dis J. 2016;35(11):1258–1261. doi:10.1097/INF.0000000000001314. 27518828

[36] Conroy DM, Williams TJ. Eotaxin and the attraction of eosinophils to the asthmatic lung. Respir Res. 2001;2(3):150–156. doi:10.1186/rr52. 11686879 PMC2002069

[37] Amerio P, Frezzolini A, Feliciani C, Verdolini R, Teofoli P, De Pita O, Puddu P. Eotaxins and CCR3 receptor in inflammatory and allergic skin diseases: therapeutical implications. Curr Drug Targets Inflamm Allergy. 2003;2(1):81–94. doi:10.2174/1568010033344480. 14561178

[38] Hoefer J, Luger M, Dal-Pont C, Culig Z, Schennach H, The JS. “Aging Factor” Eotaxin-1 (CCL11) Is Detectable in Transfusion Blood Products and Increases with the Donor’s Age. Front Aging Neurosci. 2017;9:402. doi:10.3389/fnagi.2017.00402. 29249965 PMC5717008

[39] Penders J, Thijs C, Vink C, Stelma FF, Snijders B, Kummeling I, Brandt PAVD, Stobberingh EE. Factors influencing the composition of the intestinal microbiota in early infancy. Pediatrics. 2006;118(2):511–521. doi:10.1542/peds.2005-2824. 16882802

[40] Thomas RH, Meeking MM, Mepham JR, Tichenoff L, Possmayer F, Liu S, MacFabe DF. The enteric bacterial metabolite propionic acid alters brain and plasma phospholipid molecular species: further development of a rodent model of autism spectrum disorders. J Neuroinflammation. 2012;9(1):153. doi:10.1186/1742-2094-9-153. 22747852 PMC3472254

[41] Finegold SM, Dowd SE, Gontcharova V, Liu C, Henley KE, Wolcott RD, Youn E, Summanen PH, Granpeesheh D, Dixon D, Liu M, Molitoris DR, Green JA. Pyrosequencing study of fecal microflora of autistic and control children. Anaerobe. 2010;16(4):444–453. doi:10.1016/j.anaerobe.2010.06.008. 20603222

