# Supplementary material for: Early life environmental exposures have a minor impact on the gut ecosystem following a natural birth
Source: Gut Microbes. 2021 Feb 2;13(1):1875797. doi: 10.1080/19490976.2021.1875797 (PMC7872070; doi:10.1080/19490976.2021.1875797)
Supplement: Supplemental Material [file KGMI_A_1875797_SM7681.zip › Supplementary information/SupplementalData.docx]

**Rural and urban soils have dissimilar physical, chemical and microbiological properties**

To understand if the simulated rural and urban environments were distinct, soil pH, and total elemental composition, bacteria and fungal hyphal content were quantified. Analytical testing revealed that rural soil had lower pH, higher bacterial cell counts, and higher hyphal lengths compared to urban soil (Table S1). In addition, 16S rRNA sequencing data showed that the relative microbial community composition of the two soils were dissimilar (Figure S1). The total bacterial richness of rural soil was higher (3,217 OTUs) compared to urban soil (2,818 OTUs). While both soils contained bacteria from phyla Planctomycetes, Proteobacteria, Acidobacteria, Verrucomicrobia, Chloroflexi and Actinobacteria, the relative abundances of these shared phyla were different. The relative abundance of bacterial phyla Verrucomicrobia, Planctomycetes and Acidobacteria was higher in rural soil compared to urban soil. Urban soil had higher relative abundance of phyla Actinobacteria, Proteobacteria, Bacteriodetes, and Chloroflexi compared to rural soil. There were also a few bacterial phyla that were unique to each soil. Phyla OD1 and AD3 were only present in rural soil. Phyla Gemmatimonadetes, Cyanobacteria and Bacteriodetes were only present in urban soil. Taken together, the rural soil was more acidic and had higher organic content, with higher abundances of bacteria and fungi, dominated by Planctomycetes and Verrucomicrobia bacterial phyla. In contrast, the urban soil was of mineral soil type with higher pH and lower bacterial and fungal abundance, co-dominated by Actinobacteria and Chloroflexi bacterial phyla. Overall, these results indicated that the two soil substrates used in the experiment were dissimilar in physical, chemical and microbiological characteristics, suggesting that the two soils simulated two different soil-enriched home birth environments.

| Table S1 Summary of physical, chemical and microbiological components of soils. | | | |
| --- | --- | --- | --- |
| **Property** | **Chute lake** | **A&W parking lot** | ***P* value** |
| Biome | Rural | Urban |  |
| Soil type | Organic | Mineral |  |
| Latitude/longitude | 49.69/-119.59 | 49.88/-119.48 |  |
| Elevation (m) | 1196.0 | 346.0 |  |
| Depth (cm) | 0-10 | 0-10 |  |
| C: N ratio | 29.14 | 18.75 |  |
| pH | 5.27 | 7.35 |  |
| bacterial cells per ml soil | 3.08X10^6^ | 5.9X10^5^ |  |
| hyphal length m/ml soil | 0.42 | 0.02 |  |
|  |  |  |  |

Figure S1. Relative abundance of soil bacterial phyla in rural soil and urban soil. Rural soil had higher abundance of bacterial phyla Verrucomicrobia, Planctomycetes and Acidobacteria relative to urban soil. Urban soil had higher relative abundance of Actinobacteria, Proteobacteria, Bacteriodetes, and Chloroflexi bacterial phyla compared to rural soil. Both soils contained Planctomycetes, Proteobacteria, Acidobacteria, Verrucomicrobia, Chloroflexi and Actinobacteria. Each bar represents the relative abundance of bacterial phyla that contribute more than 2% of the relative abundance of total soil OTUs of at Chute Lake rural soil and A&W parking lot urban soil.

Figure S2. Effect of early environmental exposure to rural soil, urban soil and no soil on local cytokine responses in neonatal mice. The relative gene expression of pro-inflammatory cytokines (IL-1β, TNF-α, IFN-γ), anti-inflammatory cytokine (IL-10), regulatory cytokine (TGF-β), and antimicrobial peptide (REG-3γ) in the colon tissue of 3-weeks old mice from rural soil (n=7), no soil (n=5) and urban soil (n=7) groups (Kruskal-Wallis, alpha=0.05). Data in box plots represent 25th and 75th percentiles, the lines within the boxes represent the median and the upper and lower whiskers extend from the hinge to the largest and smallest value respectively no further than 1.5*IQR (where IQR is the inter-quartile range, or distance between the first and third quartiles). In each plot, the number of mice is given by circular symbols, which sometimes superimpose on each other. The values were calculated relative to the expression of 18s with formula ΔΔC_t_.

Figure S3. Effect of early environmental exposure to rural soil, urban soil and no soil on serum cytokine and chemokine responses in neonatal and mature mice. Serum cytokine concentrations (pg/mL) were measured using Mouse cytokine/chemokine array 31-Plex (Eve Technologies). The upper detection limit for IL-13 was 40,000 pg/mL, and for all other analytes in the array it was 10,000 pg/mL. The minimum detectable concentration was 1.8 pg/mL for Eotaxin, 1.7 pg/mL for G-CSF, 1.1 pg/mL for IL-6, 7.8 pg/mL for IL-13, 10.3 pg/mL for IL-1a, 5.4 pg/mL for IL-1b, 2.0 pg/mL for IL-10, 6.7 pg/mL for MCP-1 and 2.3 pg/mL for TNF-alpha. Data in box plots represent 25th and 75th percentiles, the lines within the boxes represent the median and the upper and lower whiskers extend from the hinge to the largest and smallest value respectively no further than 1.5*IQR (where IQR is the inter-quartile range, or distance between the first and third quartiles). In each boxplot, the number of mice is given by circular symbols, which sometimes superimpose on each other (n=3-11 per group). (Wilcoxon rank-sum test; * p<0.05).

Table S2 Living environment and diet of mice in each exposure group.

| Group/treatment | Rural soil | Urban soil | No soil |
| --- | --- | --- | --- |
| Diet | SPF diets | SPF diets | SPF diets |
| Water | Sterilized tap water | Sterilized tap water | Sterilized tap water |
| Cage density | ≦5 | ≦5 | ≦5 |
| Room conditions | Specific pathogen-free animal room | Specific pathogen-free animal room | Specific pathogen-free animal room |
| Room temperature | 22**±**2**°**C | 22**±**2**°**C | 22**±**2**°**C |
| Location | CDM, same room and rack | CDM, same room and rack | CDM, same room and rack |
| Bedding | 500ml of top soil collected from Chute Lake site with sterilized bedding (aspen chips) | 500ml of top soil collected from A&W parking lot with sterilized bedding (aspen chips) | Sterilized bedding (aspen chips) |
| P0 mice | 7 weeks old (at breeding) | 7 weeks old (at breeding) | 7 weeks old (at breeding) |
| F1 generation | Colon microbiota analysis at age 3-weeks and 6-weeks based on high-throughput sequencing of 16S rDNA | Colon microbiota analysis at age 3-weeks and 6-weeks based on high-throughput sequencing of 16S rDNA | Colon microbiota analysis at age 3-weeks and 6-weeks based on high-throughput sequencing of 16S rDNA |
|  |  |  |  |
