# Supplementary figures and images for: Early life environmental exposures have a minor impact on the gut ecosystem following a natural birth
Source: Gut Microbes. 2021 Feb 2;13(1):1875797. doi: 10.1080/19490976.2021.1875797 (PMC7872070; doi:10.1080/19490976.2021.1875797)

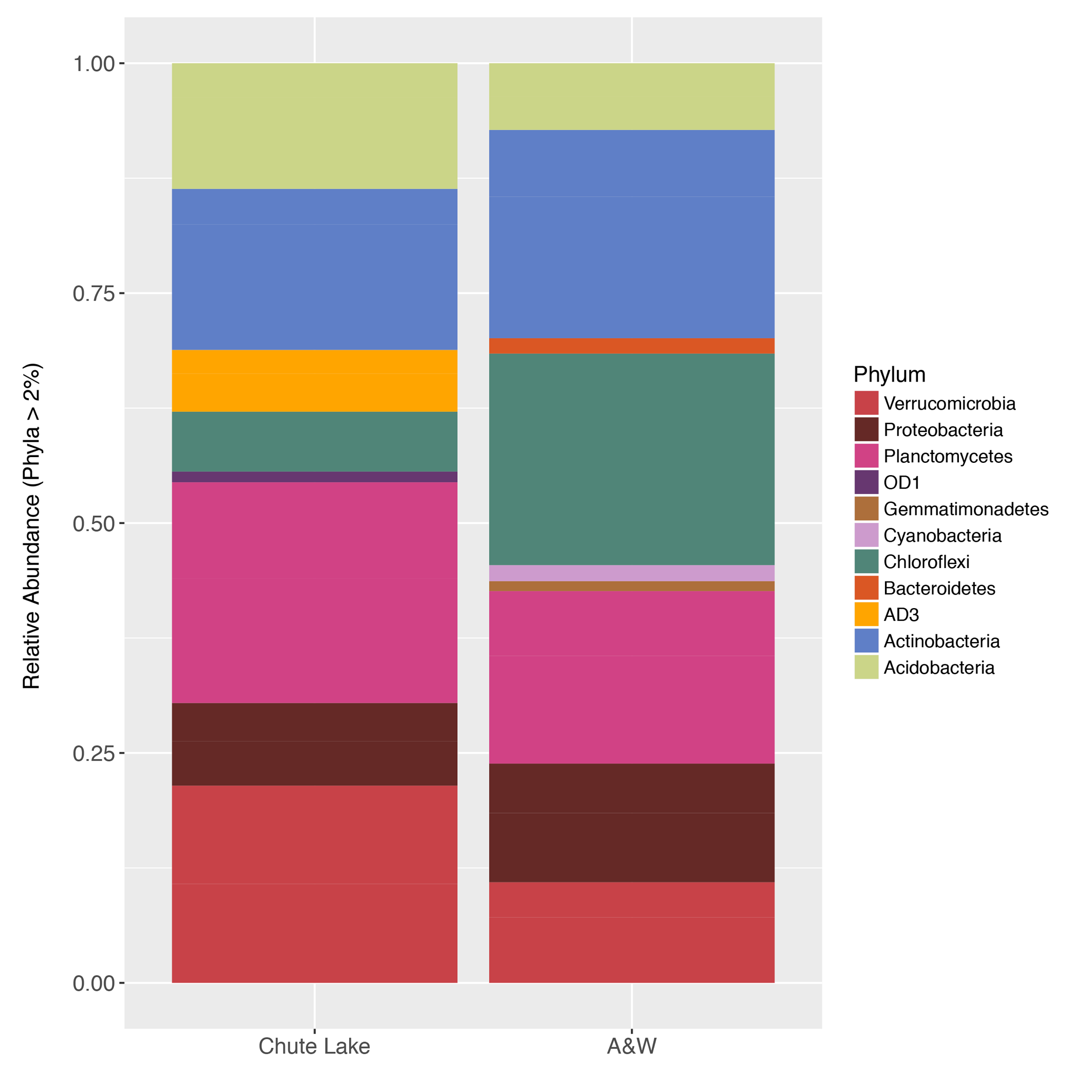

Supplement: Supplemental Material [file KGMI_A_1875797_SM7681.zip › Supplementary information/FigureS1.tif]

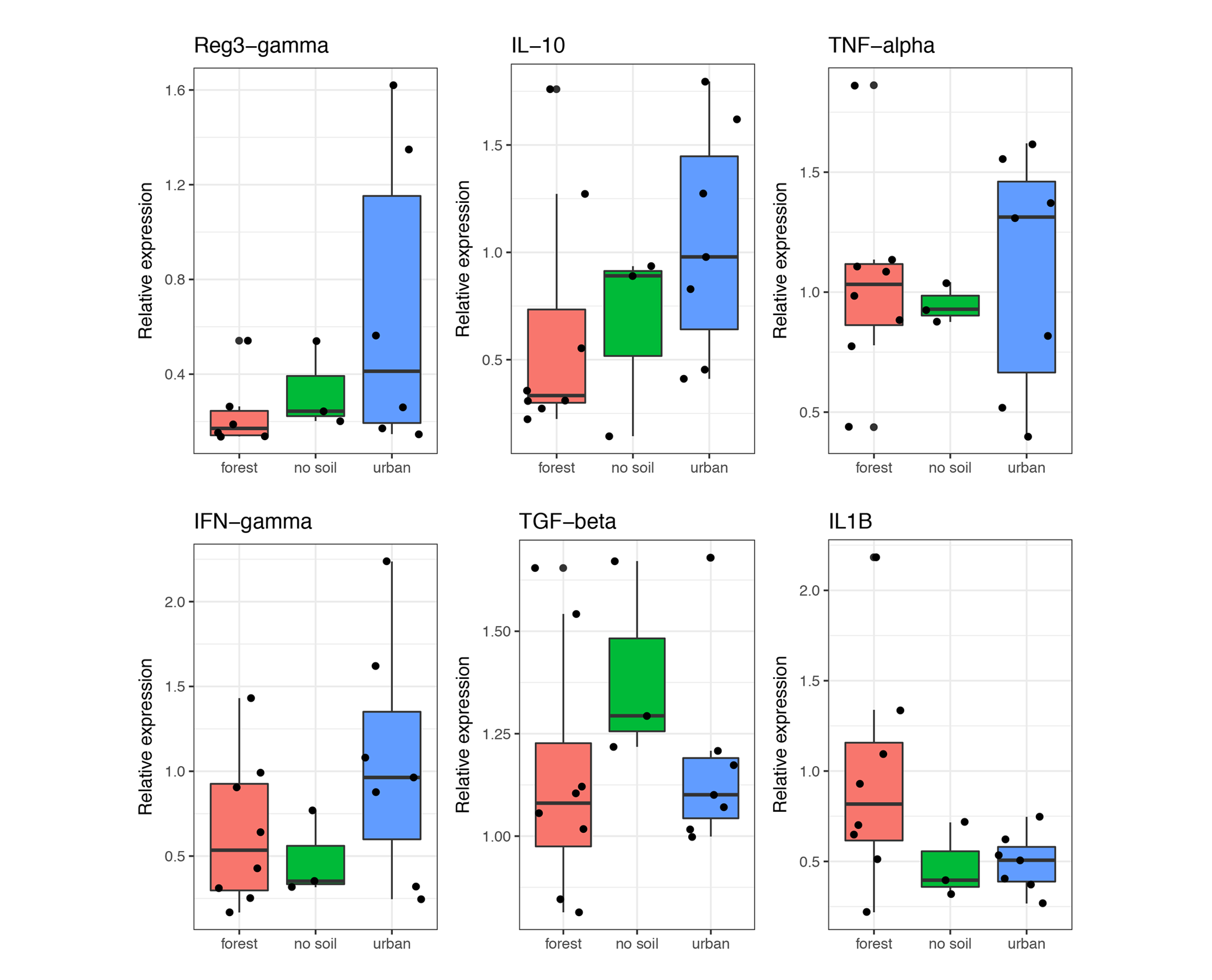

Supplement: Supplemental Material [file KGMI_A_1875797_SM7681.zip › Supplementary information/FigureS2.tif]

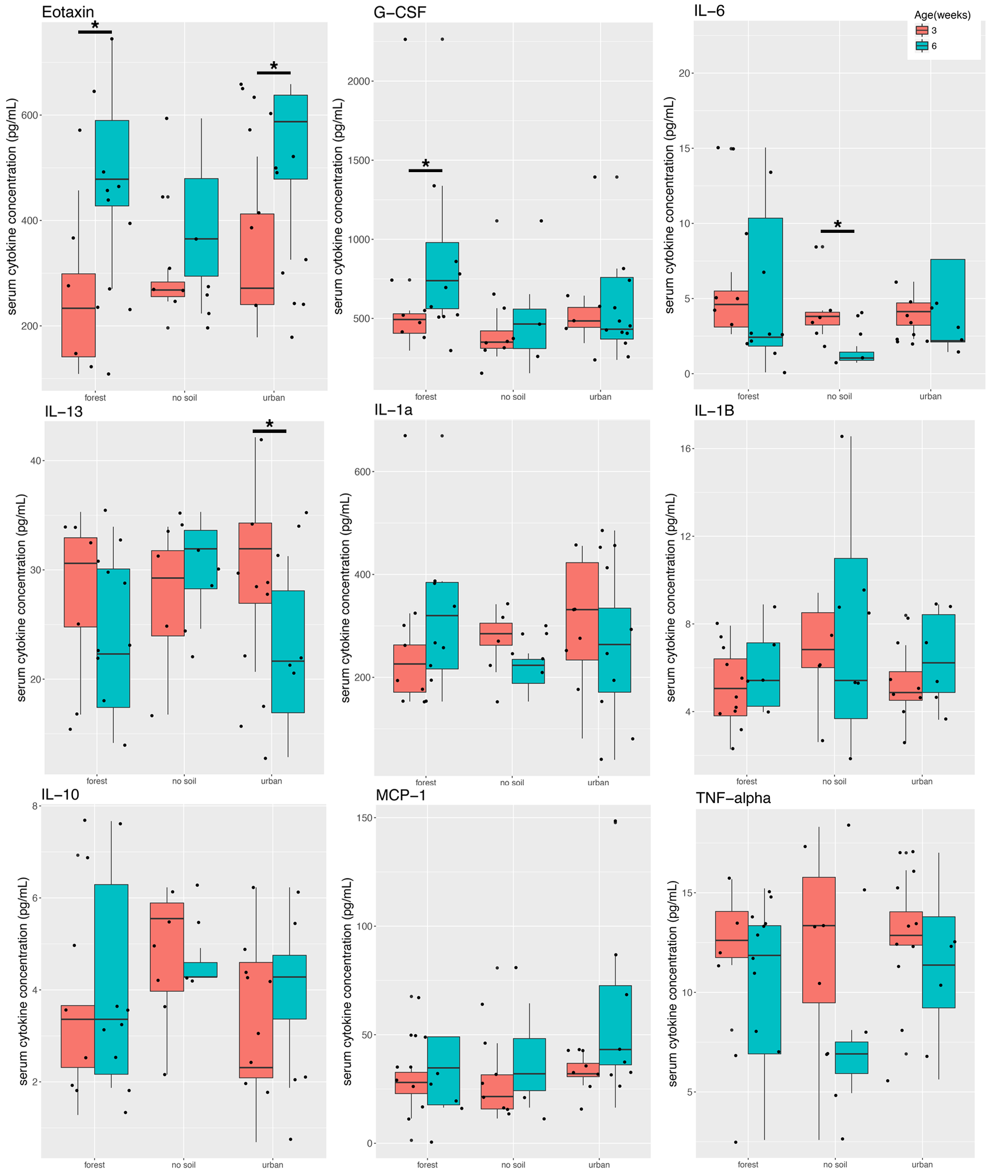

Supplement: Supplemental Material [file KGMI_A_1875797_SM7681.zip › Supplementary information/FigureS3.tif]
